# Sequential pseudoallogeneic CAR20/22/19 T-cell therapy in patient with diffuse large B-cell lymphoma relapse after allo-HSCT: a case report

**DOI:** 10.3389/fimmu.2025.1687478

**Published:** 2025-12-05

**Authors:** Yu Xiong, Shaomei Feng, Weicheng Liu, Qinlong Zheng, Biping Deng, Zhihui Li, Defeng Zhao

**Affiliations:** 1 Department of Radiation and Medical Oncology, Zhongnan Hospital of Wuhan University, Wuhan, China; 2 Hubei Key Laboratory of Tumor Biological Behaviors, Zhongnan Hospital of Wuhan University, Wuhan, China; 3 Hubei Clinical Cancer Study Center, Zhongnan Hospital of Wuhan University, Wuhan, China; 4 Department of Lymphoma and Myeloma, Beijing GoBroad Boren Hospital, Beijing, China; 5 Department of Colorectal and Anal Surgery (Clinical Center for Pelvic Floor Surgery), Zhongnan Hospital of Wuhan University, Wuhan, China; 6 Hubei Key Laboratory of Intestinal and Colorectal Diseases, Zhongnan Hospital of Wuhan University, Wuhan, China; 7 Clinical Center of Constipation and Pelvic Floor Disease of Wuhan, Wuhan, China; 8 Clinical Center of Intestinal and Colorectal Diseases of Hubei Province, Wuhan, China; 9 Quality Control Center of Colorectal and Anal Surgery of Health Commission of Hubei Province, Wuhan, China; 10 Molecular Diagnosis Laboratory, Beijing GoBroad Boren Hospital, Beijing, China; 11 Cytology Laboratory, Beijing Gobroad Boren hospital, Wuhan, China; 12 Department of Bone Marrow Transplantation, Beijing GoBroad Boren Hospital, Beijing, China

**Keywords:** refractory/relapsed diffuse large B-cell lymphoma, pseudoallogeneic CAR-T, cytokine release syndrome, autologous stem cell transplantation, allogeneic hematopoietic stem cell transplantation

## Abstract

**Purpose:**

The prognosis for patients with refractory/relapsed diffuse large B-cell lymphoma (R/R DLBCL) is dismal. Recurrence in R/R DLBCL is mostly determined by antigen loss or mutation and the limited *in vivo* survival of chimeric antigen receptor (CAR) T cells.

**Methods:**

A 38-year-old female patient was diagnosed with left breast DLBCL in March 2018. After undergoing immunochemotherapy, autologous stem cell transplantation, and radiotherapy, she relapsed in May 2019. The peripheral blood (PB) morphology showed that 36% of cells were classified as unknown. The bone marrow (BM) smear showed 71% of abnormal lymphocytes. BM flow cytometric (FCM) analysis revealed 70.24% abnormal phenotype of mature B lymphocytes. The patient’s abnormal karyotype was complex, and the 17th chromosome was missing. The p53 gene deletion (which accounted for approximately 82%) was revealed by fluorescence *in situ* hybridization (FISH) investigation.

**Results:**

Autologous CD19 CAR T cells were infused after lymphodepletion chemotherapy with cyclophosphamide and fludarabine. The patient experienced Grade I cytokine release syndrome (CRS) and achieved complete remission (CR). The genetic susceptibility gene test results suggested that the patient had potential susceptibility gene mutations for hematological tumors; therefore, allogeneic hematopoietic stem cell transplantation (allo-HSCT) was conducted as consolidation therapy. Unfortunately, the patient relapsed 5 months after allo-HSCT. Then, the patient received sequential pseudoallogeneic CAR20/22/19 T-cell therapy. The patient is currently at 4 years after allo-CAR-T treatment with BM morphology CR, negative minimal residual disease, complete donor chimerism, and no graft versus host disease (GVHD).

**Conclusion:**

Our findings suggest that pseudoallogeneic CAR-T therapy was safe and effective in patients with DLBCL who experienced relapse after allo-HSCT. Sequential administration of CAR20/22/19 T cells may have reduced the antigen escape relapse in DLBCL. For patients with DLBCL relapse after allo-HSCT, larger trials are required to validate the safety and effectiveness of pseudoallogeneic CAR-T therapy as well as its ability to lower the rate of antigen escape relapse.

## 1 Introduction

The prognosis for patients with refractory/relapsed diffuse large B-cell lymphoma (R/R DLBCL) is dismal; the median overall survival (OS) is 6 months, and the overall response rate (ORR) and complete remission (CR) are 26% and 7%, respectively (1). After salvage therapy, autologous stem cell transplantation (ASCT) and high-dose chemotherapy have become the usual treatments for R/R DLBCL patients with chemosensitive illness. However, only roughly 50% of patients experience lasting remissions following consolidative ASCT (2), and patients with aggressive subtypes such as activated B-cell (ABC)-like subtype (3), double-expressor lymphomas, and double/triple hit lymphoma (DHL/THL) (4, 5) have worse results with ASCT.

Chimeric antigen receptor (CAR) T-cell treatment targeting CD19 may be an option for patients who are not cured with ASCT, are ineligible for ASCT because of age and/or comorbidities, or have chemo-refractory illness following salvage chemotherapy (6). Regardless of the DHL/THL status or the cell of origin [ABC vs. germinal center B-cell-like (GCB) subtypes], responses were observed. Even though CAR T-cell therapy has a high rate of CR, only 30%–40% of patients experience long-lasting remissions (7). Allogeneic hematopoietic stem cell transplantation (allo-HSCT) is advised as consolidation therapy to extend lasting remission due to the elevated risk of relapse following CAR-T (8).

**Figure 1:**
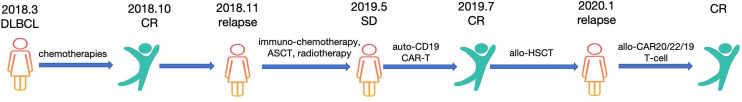
Clinical course of the patient. ASCT, autologous stem cell transplantation; auto, autologous; CAR-T, chimeric antigen receptor T cell; CR, complete remission; DLBCL, diffuse large B-cell lymphoma; SD, stable disease.

Allogeneic CAR-T could be produced by differentiating hematopoietic stem cells into the T lineage. T cells obtained from an allo-HSCT donor are known as “true” allo-CAR T cells, while T cells obtained from an allo-HSCT recipient are known as “pseudoallogeneic” CAR T cells (9–11). There are a few reports regarding pseudoallogeneic CAR-T therapy for patients with B-cell non-Hodgkin lymphoma (NHL) who experience relapse after allo-HSCT (12, 13). Herein, we describe a patient with R/R DLBCL who underwent sequential pseudoallogeneic CAR20/22/19 T-cell therapy after relapsing following immunochemotherapy, ASCT, radiotherapy, autologous CD19 CAR-T (auto-CD19 CAR-T), and allo-HSCT. Our goal in this case study was to show that consecutive pseudoallogeneic CAR20/22/19 T-cell treatment is safe and effective and reduces antigen escape relapse in patients with R/R DLBCL who experienced a relapse following allo-HSCT (**Figure 1**).

## 2 Case presentations

### 2.1 Patient characteristics before CAR T-cell therapy

**Figure 2:**
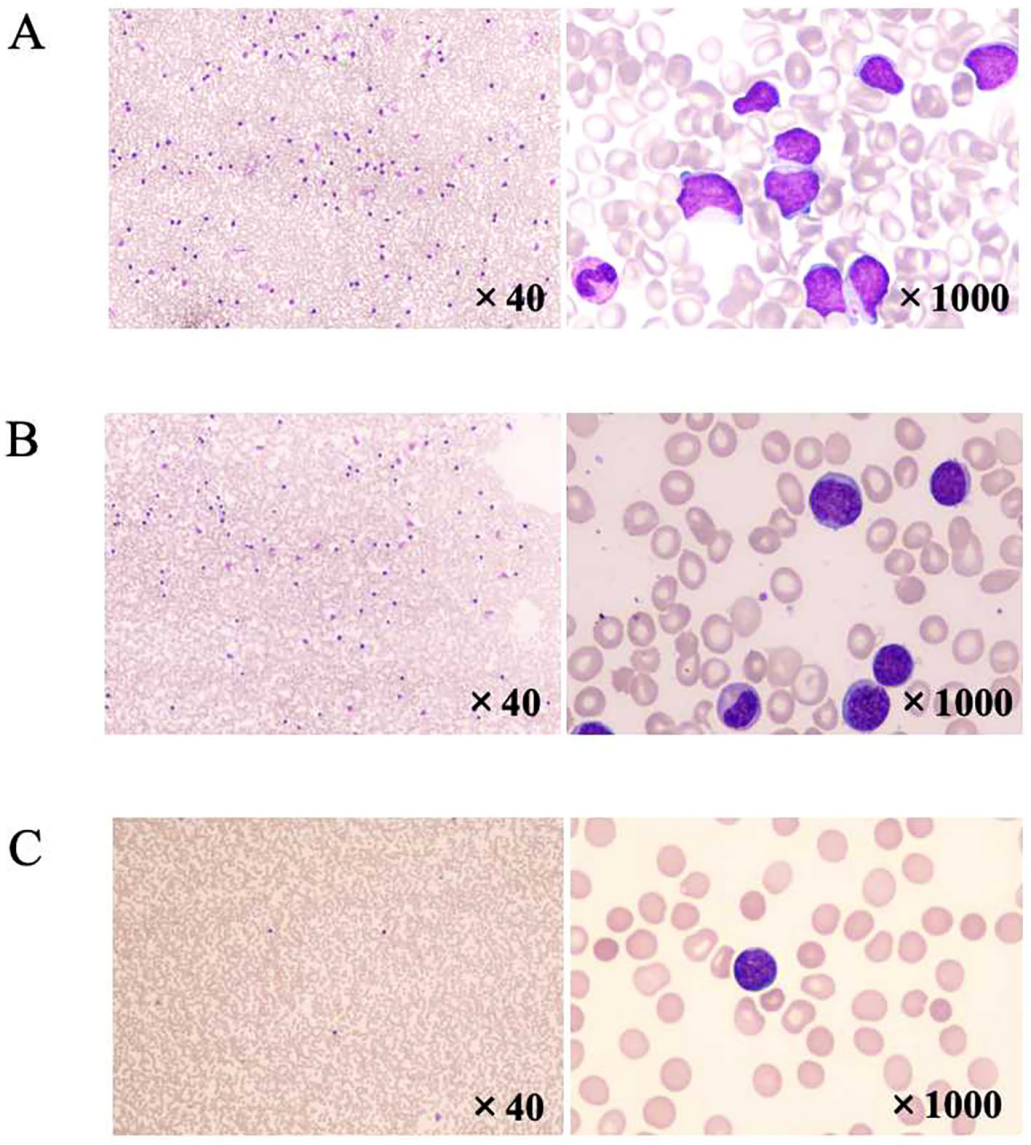
Morphological analysis. **(A)** PB morphology showed an increase in the proportion of white blood cells, with 36% of cells classified as unknown. These cells had slightly larger cell bodies; round, oval, or irregular nuclei; abundant cytoplasm; blue staining; and visible nucleoli. **(B)** BM smear showed active BM proliferation, with 71% of abnormal lymphocytes, consistent with the BM image of lymphocytic leukemia. **(C)** PB smear showed a decrease in tumor burden (abnormal lymphocytes accounted for 9%) after treatment with l-asparaginase, dexamethasone, vindesine, and BCL-2 inhibitor (venetoclax). BM, bone marrow; PB, peripheral blood.

**Figure 3:**
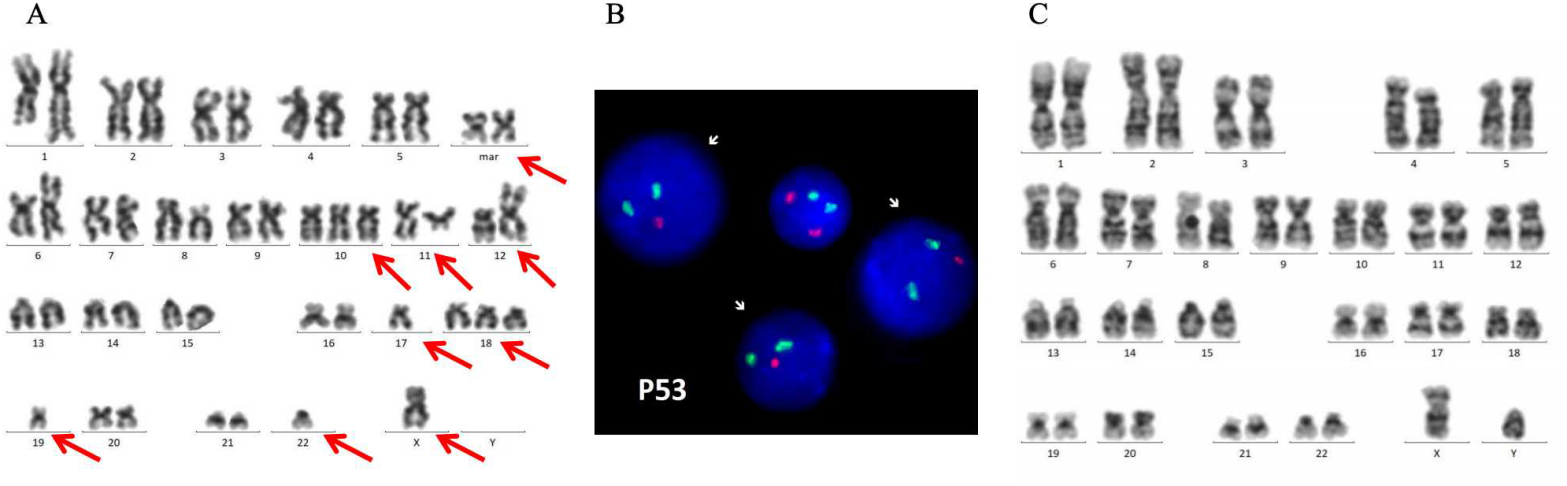
**(A)** Karyogram from the metaphase spread of the patient’s bone marrow. 46,X,-X,dup(1)(q21q32),add(6)(p25),del(8)(p22),+10,?t(11;12)(q12;p13),-17,+18,-19,-22,+2mar[5]/47,idem,+20[1]/46,X,-X,dup(1)(q21q32),+4,add(6)(p25),del(8)(p22),-17,+18,-19,-22,+2mar[2]/47,X,-X,dup(1)(q21q32),-4,add(6)(p25),+7,-17,+18,+18,-19,-11,+3mar[1]/45,X,-X,dup(1)(q21q32),add(6)(p25),del(8)(p22),-17,+18,-21,-22,+2mar[2]/46,XX (13). **(B)** p53 gene deletion (accounted for approximately 82%) was revealed by fluorescence *in situ* hybridization investigation. **(C)** Chromosome karyotype showed the donor’s karyotype after allogeneic hematopoietic stem cell transplantation.

A 38-year-old female patient was diagnosed with left breast DLBCL in March 2018. After undergoing immunochemotherapy, ASCT, and radiotherapy, she relapsed and visited our hospital in May 2019. The peripheral blood (PB) morphology showed an increase in the proportion of white blood cells, with 36% of cells classified as unknown. These cells had slightly larger cell bodies; round, oval, or irregular nuclei; abundant cytoplasm; blue staining; and visible nucleoli (**Figure 2A**). The bone marrow (BM) smear showed active BM proliferation, with 71% of abnormal lymphocytes, consistent with the BM image of lymphocytic leukemia (**Figure 2B**). BM flow cytometric (FCM) analysis revealed 70.24% (of all cells) of CD19+/CD45+ cells expressed CD22, CD79b, CD10, Kappa, and HLA-DR; partially expressed CD20 and CD11c; but did not express CD5, Lambda, CD34, TdT, CD38, CD200, CD43, CD25, CD30, FMC7, CD103, CD7, CD62L, and CD11b, Ki-67 (21.73%+), indicating an abnormal phenotype of mature B lymphocytes. Cytogenetic studies showed 46,X,-X,dup(1)(q21q32),add(6)(p25),del(8)(p22),+10,?t(11;12)(q12;p13),-17,+18,-19,-22,+2mar[5]/47,idem,+20[1]/46,X,-X,dup(1)(q21q32),+4,add(6)(p25),del(8)(p22),-17,+18,-19,-22,+2mar[2]/47,X,-X,dup(1)(q21q32),-4,add(6)(p25),+7,-17,+18,+18,-19,-11,+3mar[1]/45,X,-X,dup(1)(q21q32),add(6)(p25),del(8)(p22),-17,+18,-21,-22,+2mar[2]/46,XX (13) (**Figure 3A**). The patient’s abnormal karyotype was complex, and the 17th chromosome was missing, which may involve the loss of the p53 gene, and the prognosis was generally poor. The p53 gene deletion (which accounted for approximately 82%) was revealed by fluorescence *in situ* hybridization (FISH) investigation (**Figure 3B**), indicating poor prognosis.

### 2.2 Autologous CD19 CAR T-cell therapy and allo-HSCT

After treatment with L-asparaginase, dexamethasone, vindesine, and a BCL-2 inhibitor (venetoclax), the PB smear showed a decrease in tumor burden (abnormal lymphocytes accounted for 9%; **Figure 2C**). Cerebrospinal fluid (CSF) morphology and FCM were both negative. In May 2019, the patient started receiving the fludarabine and cyclophosphamide (FC) regimen for lymphodepletion. Three days later, the patient received auto-CD19 CAR-T cell (produced in-house, 5 × 10^5^/kg in total, 79.4% CD3+ T cells, 25.0% CD3+ CAR T cells) treatment. Following infusion, CAR+ T cells increased, peaked on day 14, and were not detectable on day 33 (**Table 1**). The course was simple, with a grade 1 cytokine release syndrome (CRS) but no immune cell-associated neurotoxicity syndrome (ICANS). Thirty days after CAR-T, PET/CT showed CR, and BM and CSF were negative. The genetic susceptibility gene testing of PB somatic cells detected TLR5, CDKN2A, and STK11 mutations. The test results suggested that the patient had potential susceptibility gene mutations for hematological tumors or immune deficiencies; her older sister carried STK11 p.F354L, CDKN2A p.L63Cfs*83, TSC1 p.Q654E, POLRMT p.Q1115Afs*45, NOTCH3 p.L1518M, FANCI p.S371G, DNAH9 p.N4073T, LRP2 p.R3305H, and C8B p.P202L heterozygous variation; her elder brother carried L1RN p.A106T, DNAH9 p.N4073T, P2RX1 p.E15K, and LRP2 p.R3305H heterozygous variation. Overall, in the absence of other donors, choosing a brother is slightly better. Therefore, a reduced-intensity conditioning (BU/Flu/ATG) allo-HSCT was conducted in July 2019. The conditioning protocol consisted of busulfan (0.8 mg/kg/q6h for 3 days), fludarabine (30 mg·m^−2^·d^−1^ for 5 days), and anti-thymocyte globulin (5 mg/kg for 4 days). After that, PB stem cells from her brother were transfused to her, containing a total of 9.07 × 10^8^/kg mononuclear cells and 12.42 × 10^6^/kg CD34+ cells. This patient successfully achieved neutrophil and platelet implantation, with neutrophil implantation time of 16 days and platelet implantation time of 14 days. Meanwhile, short-term methotrexate with cyclosporine and mycophenolate mofetil was administered to prevent graft versus host disease (GVHD). During this period, there was skin rejection, which improved after adjusting the immune suppression.

**Table 1:** Characteristics of infused CAR T cells.

Targets of CAR-T	Cell sources of CAR-T	Lymphocyte depletion regimen	Cell dose of CAR-T cell/kg	Peak day of CAR T cells (days)	CAR-T persistence (months)	CRS symptoms
CD19	Autologous	FC	5 × 10^5^/kg	14	1	Fever
CD20	Pseudoallogeneic	FC	1.6 × 10^5^/kg	13	5	Fever
CD22	Pseudoallogeneic	FC	1.16 × 10^4^/kg	–	–	No
CD19	Pseudoallogeneic	FC	3.23 × 10^4^/kg	–	–	No

CAR-T, chimeric antigen receptor T cells; CRS, cytokine release syndrome; FC, fludarabine + cyclophosphamide.

### 2.3 Allogeneic CAR T-cell therapy

In January 2020, PET/CT re-examination showed increased metabolism nodules in both breasts, which were newly developed and considered to be lymphoma recurrence. Subsequently, a pathological diagnosis of DLBCL was made via left breast biopsy. BM and CSF examinations were both negative. The chromosome karyotype showed the donor’s karyotype (**Figure 3C**), and the BM examination indicated complete donor chimerism. In March 2020, the patient started receiving the FC regimen for lymphodepletion. Three days later, the patient received pseudoallogeneic CD20 CAR-T cell (T cells harvested from the allo-HSCT recipient) (produced in-house, 1.6 × 10^5^/kg in total, 82.7% CD3+ T cells, 9.75% CD3+ CAR T cells) treatment. Following infusion, CAR+ T cells increased, peaked on day 13, and were detectable until day 149 (**Table 1**), with a grade 1 CRS but no ICANS. Thirty days after pseudoallogeneic CD20 CAR-T, PET/CT showed CR, and BM and CSF were negative.

In 2021 and 2022, sequential pseudoallogeneic CD22 CAR-T cell (produced in-house, 1.16 × 10^4^/kg in total, 76.6% CD3+ T cells, 3.46% CD3+ CAR T cells) and pseudoallogeneic CD19 CAR-T cell (produced in-house, 3.23 × 10^4^/kg in total, 72.8% CD3+ T cells, 8.15% CD3+ CAR T cells) (**Table 1**) were administered to overcome interpatient variability in antigen expression, reducing the risk of relapse mediated by antigen downregulation or loss. The course was simple, with no CRS and no ICANS. The patient returned home 1 week after receiving CAR-T infusion, so the amplification of CAR-T in the body was not monitored. The patient is currently at 4 years after allo-CAR-T treatment with BM morphology CR, negative minimal residual disease, complete donor chimerism, and no GVHD.

## 3 Discussion and conclusion

As far as we know, this is the first case of R/R DLBCL experiencing complete and long-lasting remission following allo-HSCT due to the sequential infusion of pseudoallogeneic CAR20/22/19 T-cell treatment. Although alloreactivity in CAR T cells derived from donor T cells can raise the risk of GVHD, certain groups have documented clinically significant responses in patients treated with pseudoallogeneic CAR T cells for recurrence after HSCT without significant GVHD (12–14). One explanation for the lack of GVHD could be that transplant recipient T cells are tolerated, which lowers their alloreactivity (9, 15). After receiving several pseudoallogeneic CAR-T infusions, our patient did not develop GVHD.

Reports on allogeneic CAR T-cell therapy for DLBCL patients after allo-HSCT are limited (12, 13). Four B-cell NHL patients who underwent pseudoallogeneic CAR-T treatment for the first time were described by Jain et al. (12). Two patients in that report obtained CR, and there was no development of significant GVHD. Seven patients with R/R large B-cell lymphoma (LBCL) who received pseudoallogeneic CAR-T treatment were described by another group (13). Of them, three and one patients experienced mild and severe acute GVHD, respectively, whereas four patients attained CR. The results suggest that pseudoallogeneic CAR-T therapy may be a safe and effective treatment option for LBCL patients who relapse following allo-HSCT, notwithstanding the small sample sizes of these publications.

Recurrence in R/R DLBCL is mostly determined by antigen loss or mutation and limited *in vivo* survival of CAR T cells (16). According to earlier research, combining antigen methods in CAR T-cell therapy can reduce the likelihood of relapse caused by antigen loss or downregulation by overcoming interpatient heterogeneity in antigen expression (17–23). In the treatment of patients with R/R B-cell malignancies, CAR T cells that target membrane antigens expressed on B-lymphoid cells, such as CD19, CD20, or CD22, have demonstrated significant therapeutic effectiveness (24). According to a prospective trial by Hu et al., 21 patients with R/R DLBCL underwent consecutive infusions of CAR19/20 T-cell treatments (16). Of the patients, 9.5% (2/21) and 0% (0/21), respectively, had grade 3 CRS and ICANS. Serious toxicity was not increased by the CD20 CAR T-cell infusion. There were no treatment-related deaths. Within 90 days following CD19 CAR T-cell infusion, 13 (61.9%) of the 21 patients experienced partial responses (PRs), while eight (38.1%) experienced CR. With a median duration to CR of 30 days, 10 of the 13 initial PR patients converted to CR after receiving subsequent treatment with CD20 CAR T-cell infusion. Sequential infusion of CAR19/20 T-cell treatments showed an excellent safety profile overall and may improve therapeutic results in the long term (16). Huang et al. described a pilot trial to assess the safety and effectiveness of sequential infusion of CAR19/22 T cells in 89 patients with R/R B-cell malignancies. The total response rate for the 38 NHL patients was 72.2% (95% CI, 54.8–85.8), while the CR rate was 50.0% (95% CI, 32.9–67.1). The median PFS was 9.9 months (95% CI, 3.3–NR), the median OS was 18.0 months (95% CI, 6.1–NR), and the median follow-up was 14.4 months (range, 0.4–27.4). During follow-up, one patient experienced an antigen-loss relapse. Of the individuals, 22.4% experienced high-grade CRS, while 1.12% experienced neurotoxicity. According to these findings, CAR19/22 T-cell sequential infusion was both safe and effective, and it is possible that it lowered the incidence of antigen escape recurrence in B-cell malignancies (21). In our case, CAR CD19 T cell is the secondary infusion, and its effect is lower than that of the first infusion. The patient is still in CR 4 years after undergoing sequential CAR20/22/19 T-cell treatment; the minimal residual disease test came out negative.

In conclusion, our findings suggest that pseudoallogeneic CAR-T therapy was safe and effective in patients with DLBCL who experienced relapse after allo-HSCT. Sequential administration of CAR20/22/19 T cells may have reduced the antigen escape relapse in DLBCL. For patients with DLBCL relapse after allo-HSCT, larger trials are required to validate the safety and effectiveness of pseudoallogeneic CAR-T therapy as well as its ability to lower the rate of antigen escape relapse.

## Data Availability

The raw data supporting the conclusions of this article will be made available by the authors, without undue reservation.
